# Synergistic Lyme Carditis and Coxsackievirus A Myocarditis Presenting With Fulminant Heart Failure

**DOI:** 10.1016/j.jaccas.2025.106410

**Published:** 2025-12-11

**Authors:** Joud Fahed, Abdul Mohammad, Wongelawit Zerihun, Rehman Afraz

**Affiliations:** a Department of Internal Medicine, Saint Agnes Hospital, Baltimore, Maryland, USA; b Ross University School of Medicine, Miramar, Florida, USA

**Keywords:** acute heart failure, cardiomyopathy, cardiovascular disease

## Abstract

**Background:**

Myocarditis typically results from single viral pathogens, most commonly Coxsackievirus B. Concomitant bacterial-viral myocarditis remains exceptionally rare.

**Case Summary:**

A healthy 28-year-old man developed progressive heart failure over 2 months, culminating in cardiogenic shock. Echocardiography revealed severe biventricular dysfunction (ejection fraction: 15%-20%) with complete heart block. Serologic testing confirmed dual infection with Lyme disease and Coxsackievirus A. Despite targeted antibiotic therapy, conduction did not recover, requiring cardiac resynchronization therapy with defibrillator implantation.

**Discussion:**

This represents the first reported case of simultaneous Coxsackievirus A myocarditis and Lyme carditis. Synergistic inflammatory responses between viral cytotoxicity and bacterial immune activation created fulminant heart failure exceeding what either pathogen typically causes alone.

**Take-Home Messages:**

Dual infectious myocarditis can produce devastating cardiac consequences through synergistic inflammatory mechanisms that exceed the effects of single pathogens. Young patients with fulminant myocarditis and conduction abnormalities warrant a comprehensive infectious evaluation for overlapping etiologies.

## Visual Summary

**Figure:**
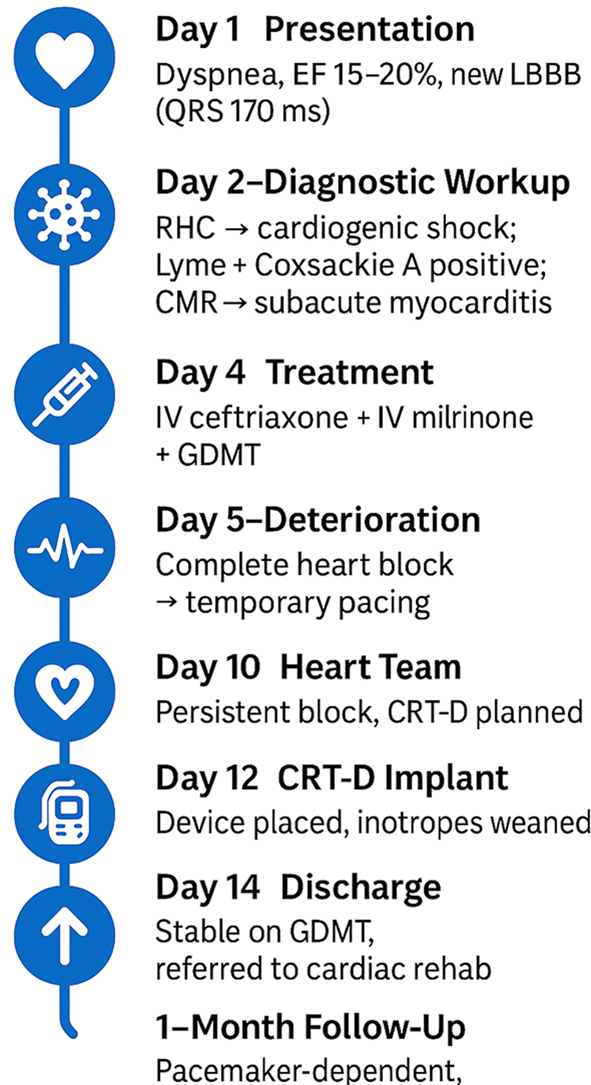

Myocarditis predominantly affects young adults and typically stems from single viral infections, with Coxsackievirus B representing the most common cause.^1^ Although Lyme disease can involve the heart through atrioventricular conduction disturbances, direct myocardial inflammation occurs infrequently.^2^ Coxsackievirus A myocarditis remains exceptionally rare, with only scattered case reports in the literature.^3^^,^^4^ We present a unique case of dual infectious myocarditis caused by concurrent Coxsackievirus A and Lyme disease, leading to fulminant cardiogenic shock and high-grade conduction disease in a previously healthy young adult.

## History of presentation

A 28-year-old previously healthy man presented to our emergency department with a 2-month history of initially mild shortness of breath during his regular jogging routine, which progressively worsened to dyspnea with minimal exertion, accompanied by orthopnea, paroxysmal nocturnal dyspnea, and bilateral lower extremity edema. He denied any known close contact with individuals experiencing febrile or infectious illnesses and reported no recent travel or animal exposures.

## Physical examination

On presentation, the patient appeared uncomfortable but not in acute distress. Physical examination was notable for bibasilar pulmonary crackles and significant bilateral pitting edema extending to the mid-calf. Cardiovascular examination revealed a regular tachycardic rhythm without murmurs, rubs, or gallops. Vital signs demonstrated sinus tachycardia at 104 beats/min with otherwise normal blood pressure and oxygen saturation.

## Past medical history

The patient had no significant past medical history, no family history of cardiomyopathy, no recent travel, and no known tick exposure.

## Investigations

Initial laboratory studies demonstrated elevated brain natriuretic peptide (759 pg/mL). High-sensitivity troponin I on arrival was within the reference range (10 ng/L) with a flat serial trend, but given the severity of left ventricular dysfunction and new conduction abnormalities, coronary angiography was pursued to exclude ischemic causes. Serum creatinine was elevated at 2.1 mg/dL, consistent with acute kidney injury.

Chest radiography showed cardiomegaly with pulmonary vascular congestion. Electrocardiography revealed sinus tachycardia, right axis deviation, new left bundle branch block (QRS interval: 170 milliseconds), and prolonged QTc interval (533 milliseconds).

**Figure 1:**
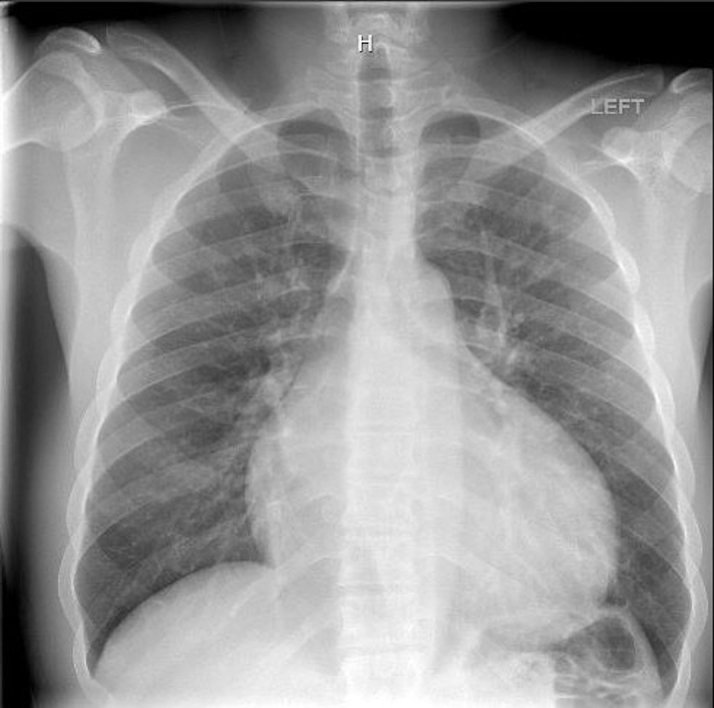
Chest X-Ray Demonstrating Cardiomegaly and Pulmonary Vascular Congestion

**Figure 2:**
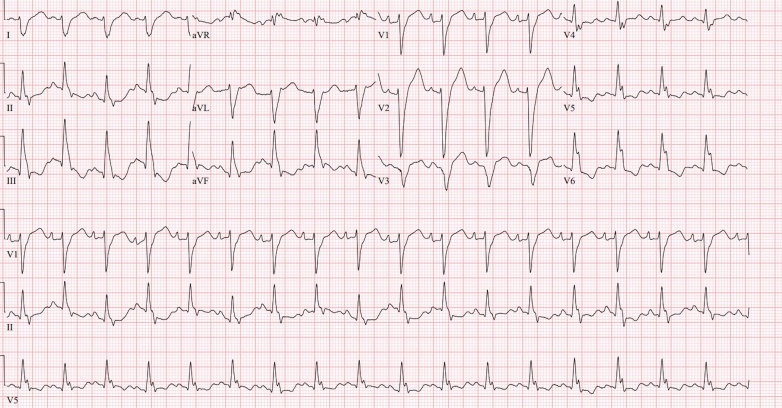
Electrocardiogram on Admission Showing Sinus Tachycardia, New Left Bundle Branch Block (QRS: 170 Milliseconds), and Prolonged QTc Interval of 533 Milliseconds

**Figure 3:**
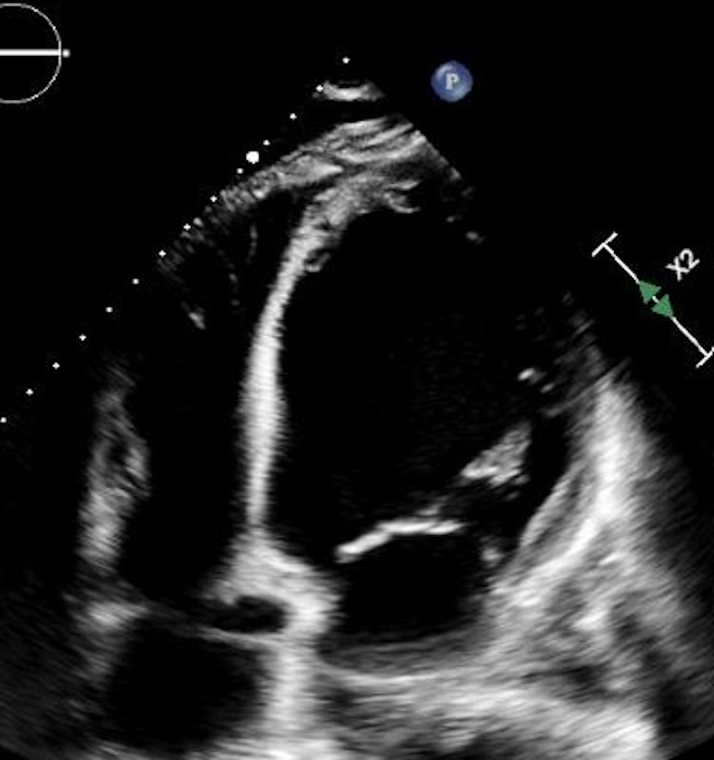
Transthoracic Echocardiogram Demonstrating Severe Biventricular Systolic Dysfunction With Global Hypokinesis and an Estimated Left Ventricular Ejection Fraction of 15% to 20%

Echocardiography demonstrated severe biventricular dysfunction with an ejection fraction of 15% to 20%, biatrial enlargement, severe mitral and tricuspid regurgitation, and estimated right ventricular systolic pressure of 60 mm Hg. Coronary angiography excluded obstructive disease. Right heart catheterization confirmed cardiogenic shock with pulmonary capillary wedge pressure of 29 mm Hg and cardiac index of 1.67 L/min/m^2^ (Figure 1, Figure 2, Figure 3).

**Figure 4:**
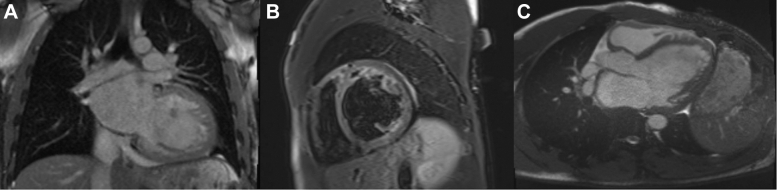
Cardiac MRI Demonstrating Sub-Acute Myocarditis With Severe Left Bi-Ventricular Dysfunction (A-C) Cardiac magnetic resonance demonstrating a markedly dilated left ventricle with severe systolic dysfunction (left ventricular ejection fraction: 12%, right ventricular ejection fraction: 12%). Left ventricular hypertrabeculation is noted, with a compacted-to-noncompacted ratio <2.3. Absence of T2 edema or late gadolinium enhancement is consistent with a subacute phase of myocarditis.

Comprehensive serologic testing revealed positive immunoglobulin M and immunoglobulin G antibodies for *Borrelia burgdorferi* and positive serology for Coxsackievirus A infection. Cardiac magnetic resonance showed left ventricular hypertrabeculation with a ratio below the diagnostic threshold for noncompaction cardiomyopathy. Notably, No T1 or T2 abnormalities were observed, suggesting a resolving rather than acute inflammatory process given the 2-month symptom duration (Figure 4).

## Differential diagnosis

Given the constellation of severe biventricular heart failure, new conduction abnormalities, and cardiogenic shock in a previously healthy young adult, fulminant giant cell myocarditis was considered due to the rapid and severe presentation, whereas cardiac sarcoidosis was contemplated but deemed less likely in the absence of systemic manifestations. Genetic or infiltrative cardiomyopathies (eg, noncompaction, storage diseases) were entertained; however, the acute timeline and absence of familial history argued against these etiologies.

The strong serologic evidence of concurrent *B burgdorferi* and Coxsackievirus A infection, along with the absence of systemic inflammatory features, ultimately guided us toward a final diagnosis of dual infectious myocarditis.

## Management

### Medical management

The patient required intensive care with intravenous milrinone for cardiogenic shock. Targeted intravenous ceftriaxone was initiated for confirmed Lyme disease. Guideline-directed medical therapy for heart failure was optimized as tolerated.

### Interventions

During hospitalization, he developed complete atrioventricular block necessitating temporary transvenous pacing. Despite adequate antimicrobial therapy, intrinsic conduction did not recover over the course of hospitalization. Because of persistent complete heart block, severely reduced ejection fraction, and elevated arrhythmic risk, a decision was made by the multidisciplinary heart team to proceed with cardiac resynchronization therapy with defibrillator implantation before discharge.

## Follow-up

The patient was discharged with close outpatient follow-up arranged for heart failure management and device monitoring. At subsequent visits, he remained pacemaker-dependent but clinically stable on optimized medical therapy. Ongoing cardiac rehabilitation and serial imaging were planned to monitor ventricular recovery.

## Discussion

To our knowledge, this case represents the first documented occurrence of concomitant Coxsackievirus A myocarditis and Lyme carditis, highlighting several important clinical and pathophysiological concepts.

### Pathophysiological Mechanisms

Coxsackievirus enters cardiomyocytes through the coxsackie-adenovirus receptor, initiating viral replication, cytolysis, and subsequent immune activation.^5^ The host response involves robust CD8^+^ T-cell activation and release of proinflammatory cytokines including interleukin-6, interleukin-1β, and tumor necrosis factor-α.^6^ Molecular mimicry between viral epitopes and cardiac proteins may perpetuate immune-mediated injury long after the acute infection.^5^^,^^6^

*B burgdorferi*, the spirochetal agent of Lyme disease, predominantly affects the cardiac conduction system, but its pathogenic effects are not limited to electrophysiology.^7^^,^^8^ It activates Toll-like receptor-2 pathways, inducing cytokine cascades and lymphocytic infiltration.^7^^,^^8^ Histopathologic studies and recent meta-analyses demonstrate that *B burgdorferi* can directly infiltrate myocardial tissue, producing interstitial edema, immune-cell recruitment, and subsequent fibrosis.^9^ These processes disrupt conduction pathways and impair contractility, whereas molecular mimicry between spirochetal and myocardial antigens sustains inflammation even after bacterial clearance.^9^

The synergistic interaction of these 2 pathogens—viral cytotoxicity and bacterial immune activation—likely amplified myocardial damage beyond what either could cause alone, resulting in profound ventricular dysfunction and irreversible conduction system injury.

### Clinical Decision-Making

This dual infectious etiology presented several diagnostic challenges. Because the patient's symptoms had been evolving for over 2 months before presentation, cardiac magnetic resonance did not demonstrate the expected T2 edema or late gadolinium enhancement typical of acute myocarditis. This highlights a key clinical principle: a normal cardiac magnetic resonance does not exclude myocarditis in subacute or resolving stages.^10^

From a therapeutic standpoint, most cases of Lyme carditis demonstrate recovery of conduction after appropriate antibiotic therapy. However, in this case, the persistence of complete heart block despite targeted antimicrobial treatment reflected irreversible damage to the conduction system, likely potentiated by the synergistic inflammatory injury of the dual infections. Early involvement of a multidisciplinary heart team facilitated timely decision-making. Cardiac resynchronization therapy with defibrillator was chosen over standard pacing to address not only conduction disease but also mechanical desynchrony and the elevated risk of malignant arrhythmias.

### Clinical Implications

This case offers several important insights for practicing clinicians. First, young patients presenting with unexplained fulminant myocarditis warrant comprehensive infectious evaluation because we learned that multiple pathogens can coexist and amplify cardiac injury in unexpected ways. Second, the timing of cardiac magnetic resonance significantly affects diagnostic sensitivity—our experience reinforces that normal findings in subacute presentations do not exclude prior myocarditis. Finally, when dual infectious processes cause irreversible cardiac damage, standard antimicrobial therapy may prove insufficient, necessitating early consideration of advanced device-based interventions.

## Conclusions

Concomitant Coxsackievirus A myocarditis and Lyme carditis, although extraordinarily rare, can produce devastating cardiac consequences through synergistic inflammatory mechanisms. This case emphasizes the importance of considering overlapping infectious etiologies in fulminant myocarditis and highlights how dual pathogen interactions can necessitate advanced therapeutic interventions beyond conventional antimicrobial treatment. Early recognition and aggressive multidisciplinary management remain crucial for optimal outcomes in these complex presentations.

**Visual Summary:**
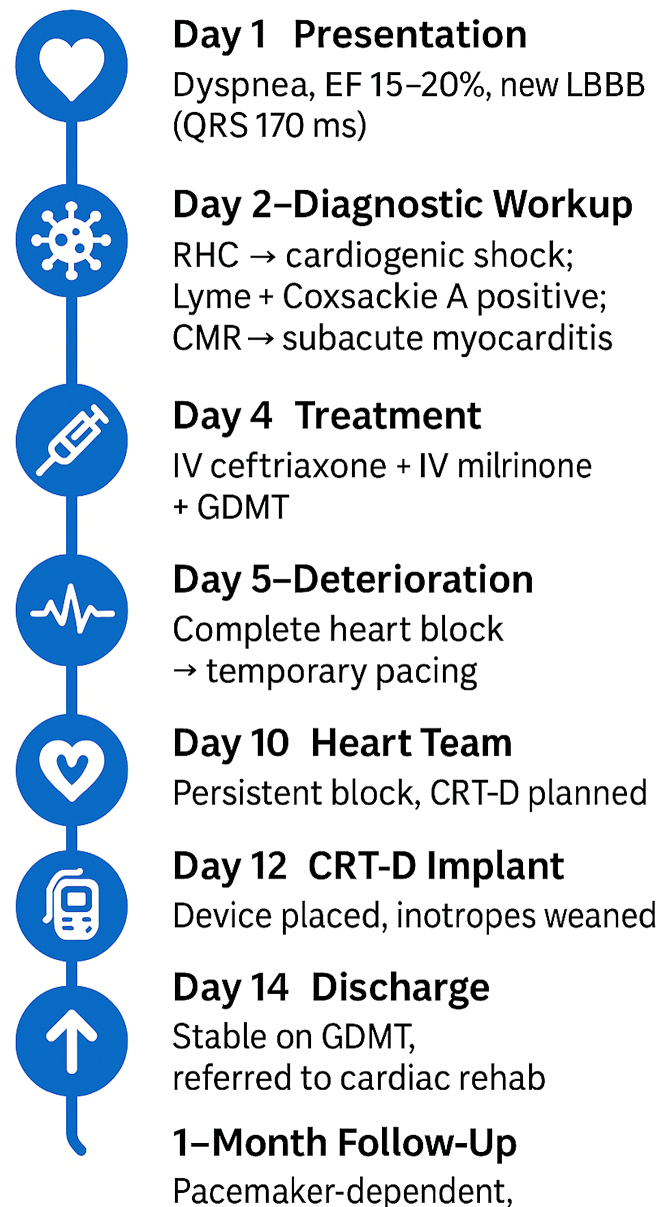
Timeline Summarizing the Presentation, Diagnostic Evaluation, Treatment, and Recovery of Our Patient CMR = cardiac magnetic resonance; CRT-D = cardiac resynchronization therapy with defibrillator; EF = ejection fraction; GDMIT = guideline directed medical therapy; IV = intravenous; LBBB = left bundle branch block; RHC = right heart catheterization.

## Funding Support and Author Disclosures

The authors have reported that they have no relationships relevant to the contents of this paper to disclose.

**Take-Home Messages**

• Dual infectious myocarditis can produce devastating cardiac consequences through synergistic inflammatory mechanisms that exceed the effects of single pathogens.
• Young patients with fulminant myocarditis and conduction abnormalities warrant a comprehensive infectious evaluation for overlapping etiologies.
